# Evaluation of the in vitro expression of ATP binding-cassette (ABC) proteins in an *Ixodes ricinus* cell line exposed to ivermectin

**DOI:** 10.1186/s13071-016-1497-2

**Published:** 2016-04-18

**Authors:** Carlo Mangia, Alice Vismarra, Laura Kramer, Lesley Bell-Sakyi, Daniele Porretta, Domenico Otranto, Sara Epis, Giulio Grandi

**Affiliations:** Department of Veterinary Sciences, University of Parma, 43126 Parma, Italy; The Pirbright Institute, Ash Road, Pirbright, Surrey GU24 0NF, Pirbright, UK; Department of Environmental Biology, University of Rome ‘La Sapienza’, Rome, Italy; Department of Veterinary Medicine, University of Bari, 70010 Bari, Italy; Department of Veterinary Science and Public Health, University of Milan, Milan, Italy; National Veterinary Institute, SVA, SE-751 89 Uppsala, Sweden

**Keywords:** ATP-binding cassette transporter, *Ixodes ricinus*, Tick cell line, in vitro, Ivermectin

## Abstract

**Background:**

Ticks are among the most important vectors of pathogens causing human and animal disease. Acaricides are used to control tick infestation, although there are increasing reports of resistance. Recently, over-expression of ATP-binding cassette (ABC) transporter proteins (P-glycoproteins, PgP) has been implicated in resistance to the acaricide ivermectin in the ticks *Rhipicephalus* (*Boophilus*) *microplus* and *Rhipicephalus sanguineus* sensu lato. Ixodid tick cell lines have been used to investigate drug resistance mechanisms. The aim of the present study was to evaluate expression of several PgPs in the *Ixodes ricinus*-derived cell line IRE/CTVM19 and to determine modulation of expression following treatment with ivermectin.

**Findings:**

IRE/CTVM19 cells were treated with different concentrations of ivermectin (0, 11, 22 or 33 μM) and incubated for 10 days. Evaluation of viability and relative expression of ABCB1, ABCB6, ABCB8 and ABCB10 genes were carried out at day 10 post treatment. Cell viability ranged between 84 % and 92 % with no significant differences between untreated and treated cells. qRT-PCR showed that ABC pump expression was not significantly modulated by ivermectin treatment. Expression of the ABCB8 PgP subfamily revealed a biphasic trend, based on the ivermectin concentration. ABCB6 and ABCB10 gene expression was not modulated by ivermectin treatment and ABCB1 expression was not detected.

**Conclusions:**

This is the first report of PgP expression in an *I. ricinus*-derived tick cell line. Development of an in vitro model for the study of acaricide resistance mechanisms would greatly facilitate screening for drug resistance in ticks.

## Findings

### Background

Ticks are among the most important vectors of a wide range of pathogens causing human and animal diseases, and several classes of acaricide are widely used to control tick infestation [1, 2]. However, there is an increasing number of reports of resistance to acaricides including macrocyclic lactones [3]. One of the most widely studied mechanisms of drug resistance is associated with the protein family of ABC transporters, which transport toxic substances outside the cell, thereby reducing their concentration inside the cell [4]. These efflux pumps are able to eliminate both endogenous and exogenous toxins and are an important “first-line” defence mechanism. Recent studies have shown that ABC transporters are present in a wide range of organisms, including mammals and arthropods, and have been implicated in drug resistance in ticks [5–7]. A recent study reported that over-expression of a gene encoding for ABC-multidrug transporters was associated with in vitro-induced resistance to ivermectin in the tick cell line BME26 [8], derived from embryos of the cattle tick *Rhipicephalus* (*Boophilus*) *microplus* [9]. In addition, ABC transporters may be involved in detoxification in the brown dog tick *Rhipicephalus sanguineus* (*sensu lato*) [10]. Despite increasing evidence that ABC transporters are likely to be involved in acaricide resistance in ticks, there have been no studies in *Ixodes ricinus*, one of the most important vectors of pathogens causing tick-borne diseases in Europe. Ixodid tick cell lines have already been used as a model for the study of drug resistance [8, 11]. The development of an in vitro model for the study of molecular resistance mechanisms and the screening of potential genetic markers of resistance in *I. ricinus* would be of great scientific interest.

The aim of the present study was to evaluate the expression of selected members of the ABC transporters subfamily B (ABCB1, ABCB6, ABCB8 and ABCB10) in vitro, following ivermectin treatment of the *I. ricinus* cell line IRE/CTVM19. Ivermectin was chosen as the test acaricide both because of its use in previous published studies on ABC transporters [6–8, 10] and because it has been shown to be active against *I. ricinus* ticks [12].

## Methods

### Reagents

All reagents were purchased from Sigma Aldrich (Milan, Italy) except where indicated.

### Cell line maintenance

Cells of the *I. ricinus* embryo-derived cell line IRE/CTVM19 [13] were seeded in flat sided 10 ml tubes (Nunc) in Leibovitz’s L-15 medium (Life Technologies, Milan, Italy) supplemented with 20 % fetal bovine serum, 10 % tryptose phosphate broth, 2mM L-glutamine, penicillin (100 U/ml) and streptomycin (100 μg/ml) and incubated at 28 °C. Medium (3/4 volume) was replaced weekly and cells were split at intervals of at least 15 days.

### Treatment of IRE/CTVM19 cells with ivermectin

IRE/CTVM19 cells seeded at a concentration of 3 × 10^6^ cells/ml in 2 ml culture medium per tube were treated immediately with different concentrations of ivermectin in 0.1 % DMSO (11 μM, 22 μM or 33 μM). Untreated cells and cells treated with 0.1 % DMSO only served as controls. Cultures were incubated for 10 days and medium was changed on the seventh day. Replacement media contained the same concentrations of ivermectin as reported above.

### Growth curve and cell viability

For growth rate analysis, four replicate tubes were used per treatment. On days 0, 5 and 10, a small aliquot of cell suspension was harvested from each tube, labelled with Trypan Blue 0.4 % w/v and counted using a haemocytometer. A test of cell viability was also performed on day 5 of treatment using the LIVE/DEAD Fixable Near-IR stain kit (Life Technologies). Cells were stained according to the manufacturer’s instructions and analysed by flow cytometry. Flow cytometry was performed using a BD FACSVerse (BD Biosciences, Stockholm, Sweden) equipped with 488 nm blue and 633 nm red lasers, and results were analysed using the FACSDiva (BD Biosciences) software. Cells frozen at -80 °C and thawed three times were used as negative controls.

### RNA extraction and determination of gene expression profile after acaricide treatment

**Fig. 1:**
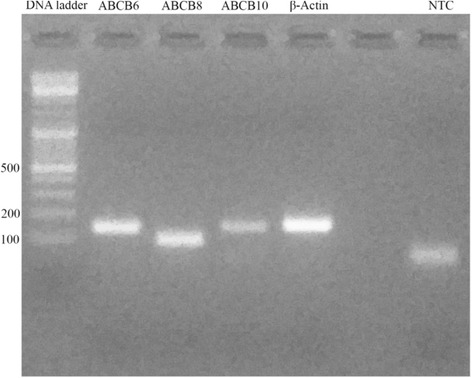
Primer couples tested in traditional PCR. All fragments were approximately 101–157 bp long. The no-template control (NTC) presented a spot due to primer dimerization

On day 10 following the start of ivermectin treatment, RNA was extracted from samples of resuspended cells from each replicate culture using an RNeasy Mini Kit (Qiagen) following the manufacturer’s instructions. RNA was measured by spectrophotometric analysis for quality and content and then converted into cDNA using a QuantiTect Reverse Transcription Kit (Qiagen). The resultant cDNAs were used as templates for molecular analysis. To date, there is no published information about *I. ricinus* sequences for any of the pumps under investigation (those encoded by the ABCB1, ABCB6, ABCB8 and ABCB10 genes); thus primers were designed (Table 1) based on conserved regions of sequences of selected ABC transporters of *I. scapularis* available in VectorBase (ABCB1: ISCW004310; ABCB6: ISCW021257; ABCB8: ISCW005908; ABCB10: ISCW008199) [14]. As an endogenous control, the *I. ricinus* β-actin gene was chosen and primers were designed based on the partial sequence available in Genbank (HQ682101). Primers were first tested in a traditional PCR using cDNA derived from an untreated control IRE/CTVM19 culture and reactions were run on a 2 % agarose gel stained with SYBR Safe Gel and examined under UV light (UView mini Transilluminator, Biorad) (Fig. 1). The amplification fragments, obtained using standard PCR conditions and the thermal profile indicated below, were sequenced in order to confirm the specificity of the amplification. The resultant sequences were deposited in the EMBL Nucleotide Sequence Database (ABCB6: LT222036; ABCB8: LT222037; ABCB10: LT222038).

**Table 1:** Primers used in the present study for evaluation of expression of the ABC subfamily B genes (ABCB1, ABCB6, ABCB8 and ABCB10) in the *Ixodes ricinus* cell line IRE/CTVM19

	Primer sequences			
ABCB1	F:	5′ –	TCTTTGCCGTCTTCTACAG	– 3′
	R:	5′ –	CAGGTTCTCTCCAGCGAT	– 3′
ABCB6	F:	5′ –	AGACTATGTCCTCTTCCTCA	– 3′
	R:	5′ –	CATCTATCACCTCTGCCTT	– 3′
ABCB8	F:	5′ –	ATCAGGAACGCCGACATC	– 3′
	R:	5′ –	AGTTTCCAGTAGACACCCTT	– 3′
ABCB10	F:	5′ –	TGTCCTAACCATTGCTCACA	– 3′
	R:	5′ –	TGATGTTCCACTAATGTCCG	– 3′
β-Actin	F:	5′ –	CACGGCATCGTGACCAACTG	– 3′
	R:	5′ –	CGAACATGATCTGAGTCATCTTCTC	– 3′

ABC-B subfamily protein expression was then evaluated by quantitative RT-PCR, using the SYBR Green master mix kit (EuroClone), according to the manufacturer’s instructions. The final concentration of each primer in all the reactions was 0.4 μM. The amplification protocol was characterised by a denaturation step (95 °C for 2 min) and 45 repeated cycles (95 °C for 10 s; 56 °C for 15 s; 72 °C for 20 s). Fluorescence signals were collected in every cycle and the presence of nonspecific products was excluded through analysis of the melting curves. Results were presented as the mean ± S.E.M. of three experiments with four replicates each, managed by CFX Manager software (Biorad) and expressed as Relative Normalised Expression (ΔΔCq).

### Data analysis

One-way ANOVA with Dunnett’s *post-hoc* test was performed using GraphPad Prism version 6(GraphPad Software, San Diego California USA, www.graphpad.com). *P*-values < 0.05 were considered statistically significant.

## Results and discussion

**Fig. 2:**
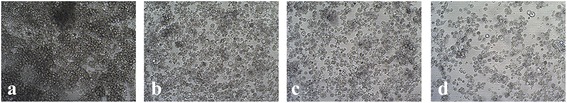
Morphology and density of IRE/CTVM19 cell line following IVM treatment. Increasing concentration of IVM (**b**: 11 μM; **c**: 22 μM; **d**: 33 μM) determined larger and more vacuolated cells compared to untreated control (**a**). Pictures were captured at 100 × magnification

**Fig. 3:**
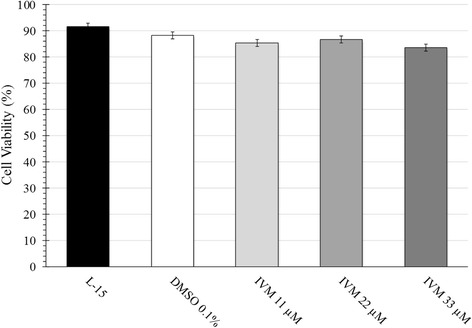
IRE/CTVM19 cell viability on day 5 of cultivation either untreated (L-15), treated with 0.1% DMSO alone (DMSO 0.1 %) or treated with ivermectin (IVM) in 0.1 % DMSO at concentrations of 11, 22 or 33 μM. Cells were evaluated by flow cytometry following Live vs. Dead® staining and data represents the mean of four replicate tubes ± S.E.M. Viability measured by Trypan Blue exclusion on days 5 and 10 was comparable (data not shown)

Growth curve analysis revealed a doubling-time of approximately ten days for IRE/CTVM19 cells under all conditions. IVM treatment modified cell morphology and adherance to the plastic tube, but did not alter cell viability (Fig. 2). As measured by Trypan Blue exclusion assay (data not shown) and flow-cytometry (Fig. 3), viability was 92 % in the untreated control cells and between 84 % and 88 % in cells treated with DMSO alone or with IVM. Differences between groups were not significant.

**Fig. 4:**
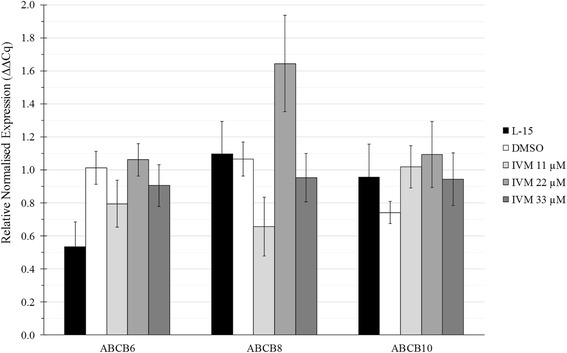
Expression of ABCB6, ABCB8 and ABCB10 genes in IRE/CTVM19 cells untreated (L-15), treated with 0.1% DMSO alone (DMSO) or treated with different concentrations of ivermectin (IVM) in 0.1% DMSO. Results were expressed as Relative Normalised Expression (ΔΔCt) vs expression of the housekeeping gene (β-Actin) and were presented as the mean ± S.E.M. of three experiments performed with four replicates each

Quantitative RT-PCR analysis showed that ABC gene expression was present in IRE/CTVM19 cells but not significantly modulated following ivermectin treatment (Fig. 4). Expression of the ABCB1 gene was not detectable at any time point in any condition (data not shown). ABCB6, ABCB8 and ABCB10 were detected, but no significant differences were seen between untreated and treated cultures or between different doses of IVM.

To the authors’ knowledge, this is the first report of expression and modulation of ABC transporters in an *I. ricinus*-derived cell line. The relative expression of ABC genes in ivermectin-treated cells ranged between 0.5 and 1.6 fold compared to the time zero control. Interestingly, ABCB8 expression showed a particular, biphasic dose-response relationship, with a low-dose stimulation and a high-dose return to the control level, as reported by Calabrese for drug-resistant vertebrate cell lines [15]. The results presented here indicate the need for further study.

Furthermore, ivermectin has been incriminated as an inhibitor of detoxification mechanisms in mammalian cell lines [16] and the results regarding ABCB8 in the present study suggest that the inhibitor effect may also be true for *I. ricinus* tick cell lines. In the only other similar in vitro study published so far [8], a clear role for ivermectin could be demonstrated both in terms of establishment of a lethal concentration 50 (LC_50_) and of a resistant tick cell sub-line. In that study, up-regulation of several ABC genes (ABCB10, ABCC1, ABCB7, ABCC2) was observed in the ivermectin-resistant cell sub-line BME26-IVM.

Interestingly, the *I. ricinus* cell line used in the present study was able to tolerate a much higher concentration of ivermectin, 30 μg/ml (33 μM), than the unselected BME26 cell line that did not survive after exposure to a concentration of ivermectin of 12.5 μg/ml or the resistant sub-line BME26-IVM for which the LC_50_ was calculated as 15.1 μg/ml [7]. Differences between the biology of the two tick species from which the cell lines were derived (*I. ricinus* and *R. microplus*), between the phenotypic composition of the cell lines themselves and/or in the culture conditions used (such as medium composition and incubation temperature) may explain the differences in the outcome of treatment and should be taken into account when refining the in vitro model of *I. ricinus*. Finally, it would be of interest to develop this in vitro model with cell lines from other economically important tick species and to evaluate the effect of different acaricides that have been reported to be losing their efficacy in the field [17].
